# Novel IL10 gene family associations with systemic juvenile idiopathic arthritis

**DOI:** 10.1186/ar2041

**Published:** 2006-09-07

**Authors:** Mark S Fife, Ana Gutierrez, Emma M Ogilvie, Carmel JW Stock, Jane M Samuel, Wendy Thomson, Lisa F Mack, Cathryn M Lewis, Patricia Woo

**Affiliations:** 1 Centre of Pediatric and Adolescent Rheumatology, Windeyer Institute for Medical Sciences, University College London, Cleveland Street, London W1T 4JF, UK; 2 ARC Epidemiology Unit, University of Manchester, Manchester, UK; 3 Guy's, Kings and St Thomas' School of Medicine, London, UK

## Abstract

Juvenile idiopathic arthritis (JIA) is the most common cause of chronic childhood disability and encompasses a number of disease subgroups. In this study we have focused on systemic JIA (sJIA), which accounts for approximately 11% of UK JIA cases. This study reports the investigation of three members of the IL10 gene family as candidate susceptibility loci in children with sJIA. DNA from 473 unaffected controls and 172 patients with sJIA was genotyped for a single nucleotide polymorphism (SNP) in IL19 and IL20 and two SNPs in IL10. We examined evidence for association of the four SNPs by single marker and haplotype analysis. Significant differences in allele frequency were observed between cases and controls, for both IL10-1082 (p = 0.031) and IL20-468 (p = 0.028). Furthermore, examination of the haplotypes of IL10-1082 and IL20-468 revealed greater evidence for association (global p = 0.0006). This study demonstrates a significant increased prevalence of the low expressing IL10-1082 genotype in patients with sJIA. In addition, we show a separate association with an IL20 polymorphism, and the IL10-1082A/IL20-468T haplotype. The two marker 'A-T' haplotype confers an odds ratio of 2.24 for sJIA. This positive association suggests an important role for these cytokines in sJIA pathogenesis.

## Introduction

Juvenile idiopathic arthritis (JIA) is the most common cause of childhood disability, with an incidence of 1 in 10 000 children under the age of 16 [1]. It is a clinically heterogeneous group of complex diseases, with polygenic and environmental factors all playing a role in aetiology. There are seven clinically distinct subtypes of JIA [2]. The most severe and least responsive to current therapies is systemic JIA (sJIA), a disease defined by a quotidian fever and one or more manifestations, including an evanescent rash, lymphadenopathy, hepatomegaly or splenomegaly, or serositis. While HLA associations are a significant genetic factor in other JIA subtypes, there are no associations with Caucasian sJIA [3]. However, a number of non-HLA genes have been reported to be associated with sJIA, including those encoding IL6 and Macrophage inhibitory factor [4,5].

IL10 is a cytokine with potent immunoregulatory and anti-inflammatory properties. It acts to suppress the release and function of a number of pro-inflammatory cytokines, including IL1, tumour necrosis factor (TNF)-α, and IL6 [6]. Low levels of IL10 production associated with autoimmune disease, such as rheumatoid arthritis, psoriasis, and collagen-induced arthritis, suggest defective regulatory roles of IL10 in limiting inflammation and reversing immunopathology. However, IL10 is also a stimulatory factor for mast cells, B cells, and thymocytes [7,8].

In the upstream regulatory region of the gene encoding IL10, the three most characterised single nucleotide polymorphisms (SNPs) are at positions -1,082 (G to A), -819 (C to T) and -592 (C to A) [9,10]. There is absolute linkage disequilibrium (LD) between the IL10-819 and IL10-592 SNPs. In the Caucasian populations only three out of the remaining four possible haplotypes occur: GCC, ACC and ATA. We have previously shown that stimulation of human whole blood cultures with bacterial lipopolysaccharide (LPS) results in a large variation between individuals in IL10 secretion. When examined in the context of IL10 genotype, it was revealed that the ATA/ATA genotype is associated with significantly lower IL10 production [11,12]. In addition, we also showed a significantly increased frequency of this low-expressing ATA haplotype in extended juvenile oligoarthritis. There is strong evidence to support genetic control of IL10 levels, with production levels showing concordance in monozygotic twins and a genetic component of over 75% [13]. Turner and colleagues [9] demonstrated that the difference in IL10 secretion is associated with the presence or absence of an 'A' at position -1,082 of the human IL10 promoter.

In the severe forms of JIA, treatment is often limited to high doses of glucocorticoids. It is of interest, therefore, that pre-treatment with IL10 improves the ability of dexamethasone in suppressing IL6 (a cytokine correlated with sJIA disease activity) in whole-blood cultures (p < 0.01) [14]. Furthermore, LPS-stimulated whole blood cultures from sJIA patients showed reduced levels of IL10 compared to healthy, age matched controls [11]. This reduced capacity of sJIA patients to produce sufficient quantities of this anti-inflammatory cytokine may suggest a pathogenic role for IL10 in this disease.

Two recently described members of the IL10 family are IL19 and IL20. The genes encoding these cytokines are located within a highly conserved cytokine gene cluster in 1q32. Like the T-Helper 2 (TH2) IL4 cytokine gene cluster, recent evidence from the mouse IL10 gene family cluster suggests that there is coordinate regulation of these cytokines by distal regulatory elements spanning the locus [15]. Both IL19 and IL20 are produced by a diverse range of cell types, including monocyte/macrophages, T- cells and keratinocytes [16]. Interestingly, long-term exposure of T cells to IL19 and IL20 down-regulated interferon-γ but up-regulated IL4 and IL13 and supported the polarisation of naive T cells to Th2-like cells [17]. Apart from significant genetic association of Hepatitis C virus clearance with IL10/19 and IL20, and psoriasis with IL19/20 and IL24, to our knowledge no additional disease association studies with these cytokine genes have been conducted [18,19].

The IL10 SNPs are located 592 base-pairs (bp) and 1,082 bp upstream of the IL10 transcription start site. The non-synonymous SNP in IL19 and a SNP in IL20 are situated further upstream at 70 kb and 93 kb, respectively, relative to the transcription start of IL10. These SNPs are all potentially functional polymorphisms and were chosen as representatives from this region of the cytokine cluster for association analysis with sJIA. SNPs with a minor allele frequency >0.10 were selected to increase the power to detect an association. This is the first association analysis of members of the IL10 gene family to be performed in sJIA.

## Materials and methods

### Patients and control sample collection

Patients' DNA from the British Paediatric Rheumatology Group (BPRG) National DNA repository held at the Arthritis Research Campaign (ARC) Epidemiology Unit, Manchester, was used for genotyping the markers across the IL10, IL19 and IL20 loci. Additional patients were also recruited from the Outpatient Departments at both Great Ormond Street Hospital and the Middlesex Hospital. Ethical approval for the study was obtained and parents gave informed consent. Two ethnically matched healthy control populations were used for this case/control study. One population was composed of first time blood donors attending the national blood transfusion centre in London (*n *= 248). The second population was collected from individuals in the 16 to 30 years age group from a GP practice in a stable population of the west Midlands (*n *= 225).

### Genotyping

DNA from 172 patients with sJIA and 473 unaffected controls was genotyped for the IL19+13735 (rs2243191) and IL20-468 (rs1400986) SNPs, and two previously characterized SNPs in IL10, IL10-592 (rs1800872) and IL10-1082 (rs1800896).

Pyrosequencing was used to genotype the polymorphisms in IL10. A fragment 50 to 200 bp flanking the SNP was PCR amplified. The anti-sense primer was biotinylated to allow the preparation of single-stranded DNA. An aliquot of 25 ng of DNA was amplified in a 25 μl PCR reaction with 0.25 μM of each primer, 0.2 mM of each of the four dNTPs, 0.5 U of Taq polymerase (Promega, Madison WI USA), 2.5 mM of MgCl_2 _and 1× KCl buffer. The cycling parameters consisted of an initial denaturation at 94°C for 5 minutes and then 35 cycles of 30 s denaturation at 94°C, 30 s annealing at 67°C and 11 s extension at 72°C. A final extension step was carried out for 7 minutes at 72°C. We immobilized 10 μl of the PCR product on streptavidin sepharose beads (GE Healthcare, Uppsala, Sweden) and the pyrosequencing was performed according to the standard PSQ HS 96A system protocol (Pyrosequencing AB, Uppsala, Sweden).

Genotyping of the SNPs in the genes encoding IL19 and IL20 was carried out using Sequenom MassARRAY, (San Diego, CA, USA). Primers designed by RealSNP assay (Sequenom) amplified approximately 100 bp of sequence surrounding the target SNP. PCR was carried out using 1× HotStar *Taq *PCR buffer 2.5 mM MgCl_2 _(Qiagen, Crawley, West Sussex, UK), 500 μM of each dNTP, 0.1 U Enzyme HotStar *Taq *polymerase (Qiagen), 100 nM primer, and 2.5 ng of genomic DNA in a total volume of 5 μl. PCR was followed by incubation with shrimp alkaline phosphatase to digest unincorporated dNTPs and primers. This product was used to carry out MassExtend reactions using flanking extend primers, dideonucleotides and Taq polymerase to extend the primer through the polymorphic site. Finally, the extended product was purified of excess ions using an ion-exchange resin. Genotypes were acquired using a chip-based matrix-assisted laser desorption ionization-time-of-flight (MALDI-TOF) mass spectrometer.

### Association analysis

Tests for Hardy-Weinberg equilibrium and for single locus association were performed using chi-squared statistics. Haploview was used to assess the level of LD between markers across this region by analysing control genotypes only [20]. We examined evidence for association of the four SNPs by single marker and haplotype analysis using Cocaphase software (UNPHASED) [21]. The p values have not been corrected for multiple testing since each SNP had a strong prior hypothesis for testing for association with systemic JIA.

## Results

### Linkage disequilibrium analysis

**Figure 1:**
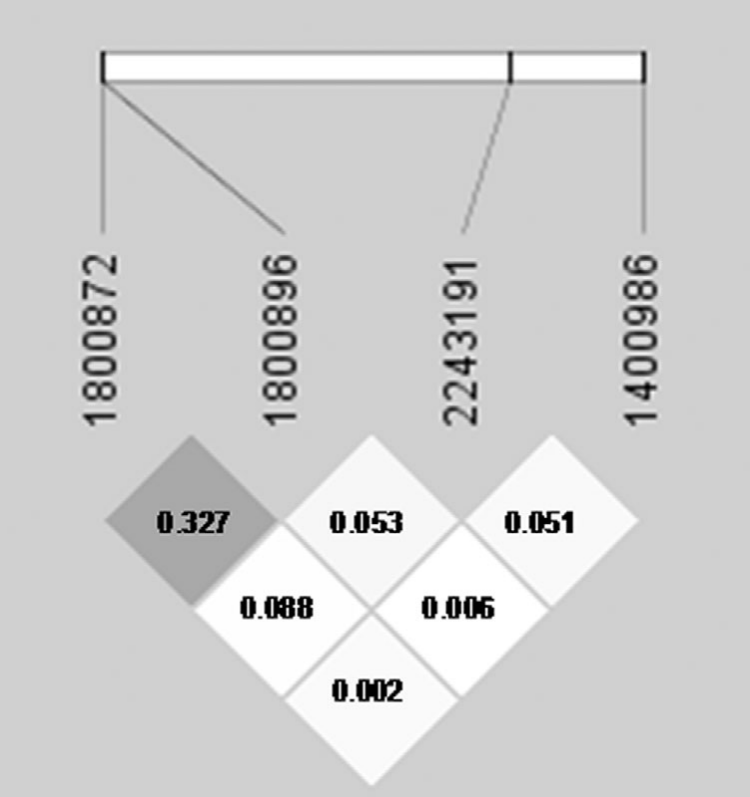
Pairwise linkage disequilibrium (r^2^) for the four markers tested in the genes encoding IL10, IL19 and IL20. The rs numbers refer to SNPs IL10-1082 (rs1800896), IL10-592 (rs1800872), IL19+13735 (rs2243191) and IL20-468 (rs1400986).

Analysis of LD across these markers confirms previous reports of an intermediate level of LD between IL10-1082 and IL10-592 within the IL10 promoter region (data not shown). No LD was observed between the IL10 markers and either IL19+13735 or IL20-468 SNPs, or between IL19+13735 and IL20-468. LD patterns using r^2 ^were similar in the cases (Figure 1).

### Association analysis

All SNPs were in Hardy-Weinberg equilibrium in cases and controls. Significant differences in allele frequency were observed between sJIA cases and controls for IL10-1082 (p = 0.031). The association was not, however, due to the ATA haplotype, as no further genotype or haplotype effects were observed with IL10-592. Significant differences in allele frequency were also observed for the IL20 marker IL20-468 (p = 0.028; Table 1). When the two associated markers were examined as a haplotype there was an increased level of significance in association with disease (global p = 0.0006). Analysis of the haplotype frequencies revealed a decrease of the common haplotype in the cases, along with an increase in the carriage of the rare haplotype containing the low expressing 'A' allele variant of IL10-1082 and the rare 'T' allele of IL20-468 (Table 2).

There was no evidence of interaction between these SNPs. Each copy of IL10-1082A confers an increased risk of 1.3 of developing sJIA, and each copy of IL20-468T confers a risk of 1.507, with a combined risk of 2.24 across both SNPs.

The IL10-592 SNP and IL19+13735 SNPs show no significant association with sJIA.

## Discussion

A feature common to all subgroups of JIA is chronic inflammation and synovitis. Cytokines are important regulators of inflammation and many studies have shown persistent cytokine imbalance in JIA [12,22,23]. Ourselves and others have previously shown that IL6 levels in the serum and synovial fluid of sJIA patients are elevated and appear to correlate with disease activity [4,24]. Furthermore, we have replicated the case/control association study of this locus with sJIA, using the transmission disequilibrium test, confirming its role in disease susceptibility [25]. The haplotypic structure of the IL6 locus was extensively examined and we showed that a four marker haplotype has greater power in demonstrating association with sJIA susceptibility [26]. As IL10 suppresses the release of pro-inflammatory cytokines such as IL6 and TNFα, it is a normal endogenous feedback factor for the control of inflammation. Having previously identified IL10 as a marker for another severe form of JIA, extended oligoarthritis, here we aim to broaden the analysis to the IL10 gene family in sJIA using haplotype analysis.

In this study we have shown a significant increased prevalence of the IL10-1082 A allele, associated with low IL10 production, in patients with sJIA. In addition, we have also shown a separate association with another member of the IL10 gene family: IL20. The significant association of the low expressing IL10 allele with sJIA implies that the regulation of pro-inflammatory cytokine production in multiple cell types, as well as the regulation of CD4+ cells, may be suboptimal. Interaction of IL10 with other cytokines in antigen presentation and T cell polarisation pathways could determine the phenotype of the disease. For example, decreased levels of IL10 concomitant with increased IL6 would tend to favour the development of Th2 T helper cells as well as preventing the differentiation of regulatory T cells (T regs) in some instances. Our earlier study of whole blood cultures of systemic JIA has shown that there is no spontaneous production of cytokines but that there may be an inadequate IL10 response to LPS stimulation compared to that of controls [11]. IL10 is produced by some T regs generated in the periphery (Tr1) in humans, as well as by regulatory B cells in animal models [27]. T regs have been found to increase after autologous stem cell transplantation and disease remission in sJIA [28]. Thus, there is a suggestion from these preliminary observations that the numbers of T regs are suboptimal in sJIA, and this may also play a role in the decreased IL10 levels in these patients.

Recent evidence indicates that IL19 may also play a central role in inflammation as it up-regulates monocyte derived IL6 and TNFα in mice [29]. Like IL20, IL19 is also associated with psoriasis, another inflammatory disease [18]. Although we show no evidence for association of IL19 in this study, it cannot be eliminated as a candidate gene for sJIA. An in-depth tagging SNP approach to refute or confirm any associations in this region is currently being planned.

The increased significance of the association with the IL10-1082/IL20-468 haplotype relative to the single markers may indicate a functional role of the compound haplotype in disease susceptibility. Alternatively, it could be that neither the IL10 nor the IL20 markers are sJIA susceptibility loci, but merely in strong LD with an as yet uncharacterised functional polymorphism. There has only been limited analysis of the regulation and expression of the cluster of IL10-like genes at this locus, but this does point to a coordinated expression of these genes. Jones and Flavell [15] identified three enhancer elements in a 40 kb region between the genes encoding IL19 and IL10. Two of these enhancer elements, located 9 kb upstream and 6.45 kb downstream of the gene encoding IL10, display cell-specific function and also exhibit basic promoter activity. Hence, any polymorphism within these novel regulatory elements may alter the function or expression of any intermediate regulatory RNAs. The association we observe for the IL10-1082/IL20-468 haplotype may be attributable to any one of these regulatory regions carried on this haplotype.

Due to the low prevalence of sJIA, the number of cases used in this study is restricted. To compensate for the limited case collection, we have used a control :case ratio of 2.75 to increase the power of the study. Additional patient samples will be needed to confirm this finding in a replication study.

## Conclusion

This study describes a new association between the two IL10 gene family members and children with sJIA, indicative of a central role for these cytokines in disease pathogenesis.

## Abbreviations

bp = base-pairs; JIA = juvenile idiopathic arthritis; LD = linkage disequilibrium; LPS = lipopolysaccharide; sJIA = systemic JIA; SNP = single nucleotide polymorphism; T regs = regulatory T cells; TH2 = T-Helper 2; TNF = tumour necrosis factor.

## Competing interests

The authors declare that they have no competing interests.

## Authors' contributions

MF conducted the genotyping using Pyrosequencing and performed the association analyses. AG helped with the genotyping. EO contributed and advised on the data analyses and helped to draft the manuscript. CS performed the Haploview analysis. JS advised on the molecular biology aspects of the project and helped to draft the manuscript. WT and LM were involved in the Sequenom genotyping. CL contributed and advised on the data analyses and helped to draft the manuscript. PW participated in the design and coordination of the study and helped to draft the manuscript.

## Figures and Tables

**Table 1:** Single marker analyses of association in systemic juvenile idiopathic arthritis

Marker	Case		Control		Odds ratio	p value
	Allele 1	Allele 2	Allele 1	Allele 2		

IL10^-1082G/A ^(allele 1 = G; allele 2 = A)	139 (0.45)	169 (0.55)	425 (0.52)	387 (0.48)	1.335	0.031
IL10^-592C/A ^(allele 1 = C; allele 2 = A)	222 (0.71)	92 (0.29)	647 (0.76)	205 (0.24)	1.308	NS
IL19^+13735C/T ^(allele 1 = C; allele 2 = T)	188 (0.77)	56 (0.23)	692 (0.78)	194 (0.22)	1.063	NS
IL20^-468C/T ^(allele 1 = C; allele 2 = T)	189 (0.79)	51 (0.21)	765 (0.85)	137 (0.15)	1.507	0.028

The numbers in the table represent the typed alleles for IL10-1082, IL10-592, IL19+13735 and IL20-468; the frequencies are in parentheses. NS, non-significant.

**Table 2:** Haplotype analysis results for association in systemic juvenile idiopathic arthritis

Haplotype (IL10^-1082^/IL20^-468^)	Cases	Controls	Chi square	p value
-1082G/-468C	118.3 (0.36)	428.4 (0.46)	10.09	0.0015
-1082G/-468T	34.2 (0.10)	70.7 (0.08)	2.431	NS
-1082A/-468C	132.3 (0.40)	364.6 (0.39)	0.2943	NS
-1082A/-468T	47.2 (0.14)	76.29 (0.08)	12.57	0.0004

The numbers in the table represent the expected number of haplotypes, inferring missing genotype data from haplotype frequencies and linkage disequilibrium. Haplotype frequencies shown in parentheses. Global significance p = 0.0006. NS, non significant.
